# The Spectrum of Echocardiographic Findings Across Stages I-V of Chronic Kidney Disease

**DOI:** 10.7759/cureus.65359

**Published:** 2024-07-25

**Authors:** Yasir Hussain, Anjum Shahzad, Samee Javed Bhatti, Adnan Ahmad Zafar, Badar U Din Shah, Muhammad Irfan Jamil, Adeel Ahmed, Ayesha Naeem Aamir

**Affiliations:** 1 Nephrology, Lahore General Hospital, Lahore, PAK; 2 Nephrology, Islam Medical College, Sialkot, PAK; 3 Nephrology, Geisinger Medical Center, Danville, USA; 4 Medicine, Lahore General Hospital, Lahore, PAK; 5 Cardiology, King Edward Medical University, Lahore, Lahore, PAK

**Keywords:** systolic and diastolic myocardial functions, systolic heart failure, left ventricular hypertrophy (lvh), echocardiogram (echo), chronic kidney disease (ckd)

## Abstract

Aim and background: Chronic kidney disease (CKD) is associated with increased cardiovascular morbidity and mortality. This study aimed to assess the frequency of cardiac abnormalities across different stages of CKD, providing insights into the relationship between renal dysfunction and cardiac abnormalities.

Material and methods: A cross-sectional observational study was conducted at Lahore General Hospital's Nephrology Department, enrolling 356 non-dialysis CKD patients (stages I-V) over one year. Participants aged 18-65 years with CKD duration of three months or more were included. Exclusion criteria encompassed dialysis dependency, transplantation, acute kidney injury, and various cardiac conditions. Detailed echocardiographic evaluation of cardiac structure and function was noted.

Results: This study included 356 patients with CKD across stages I-V, with the majority in stages III (44.7%) and IV (36.5%). Significant variations were observed in age (p<0.000), hypertension prevalence (p=0.004), and smoking status. Haemoglobin, calcium, and phosphate levels differed significantly across stages (p<0.001). Echocardiographic findings revealed significant differences: left ventricular hypertrophy frequency increased from 12.5% in stages I-II to 60.0% in stage V (p=0.001); diastolic dysfunction worsened, with grades 2-3 dysfunction increasing from 6.2% in stages I-II to 51.4% in stage V (p=0.000); systolic dysfunction increased with reduced ejection fraction (<45%) more common in advanced stages (p=0.000); global longitudinal strain worsened from -18.47% to -15.34% (p=0.000); left atrial volume index and pulmonary hypertension also increased significantly (p=0.049).

Conclusion: This study demonstrates a significant correlation between the progression of CKD and the severity of echocardiographic abnormalities. As CKD advances, structural and functional cardiac abnormalities increase, underscoring the importance of early cardiac evaluation and intervention to improve cardiovascular outcomes in non-dialysis-dependent CKD patients.

## Introduction

Chronic kidney disease (CKD) prevalence varies geographically. Its worldwide estimated prevalence is 13% [1]. ​ In Pakistan, the prevalence of CKD ranges from 12.5% to 21.2%, with diabetic nephropathy (27.1%) as the leading cause. Other significant etiologies include CKD of unknown aetiology (16.6%), hypertensive nephropathy (15.2%), chronic glomerulonephritis (14.4%), and renal stone disease (12.5%) [2]. These metrics highlight the importance of addressing CKD and its underlying causes to manage and reduce the disease burden effectively.

Cardiovascular disease (CVD) is one of the most common complications. CKD patients encounter increased risk of hospitalization, morbidity and mortality due to cardiac problems i.e., myocardial infarction and heart failure. Although CKD is an independent risk factor for CVD, it is also known that heart disease can affect renal function, leading to the progression of CKD [3,4]. Concurrently, metabolic and haematological abnormalities of CKD, such as anaemia, calcium-phosphate imbalances, dyslipidemia and hyperuricemia, make structural and functional alterations in the heart through various mechanisms [5].

Transthoracic echocardiography (TTE) is a non-invasive, cost-effective, and widely available imaging modality used to evaluate structural and functional components of the heart. By evaluating ventricular structural changes, systolic and diastolic function, valvular abnormalities, and pulmonary pressures, it provides routine cardiovascular assessment of patients. TTE can detect subclinical cardiac changes that may precede overt CVD, offering a window into the early stages of cardiomyopathy in CKD patients [6,7].

Previous studies have focused spectrum of echocardiographic abnormalities in dialysis-dependent end-stage kidney disease (ESKD) patients; it is essential to recognize these changes in non-hemodialysis CKD patients [8-10]. This emphasises the importance of timely recognition of patients at risk of cardiac diseases. Early identification and intervention of cardiac complications in CKD patients are necessary for a better prognosis. However, a clear understanding of the spectrum of these abnormalities in non-hemodialysis CKD patients remains limited. This study aimed to determine the cardiac structural and functional abnormalities among patients of CKD stages I-V.

## Materials and methods

A cross-sectional observational study was conducted at the Nephrology Department of Lahore General Hospital, Lahore, following ethical approval from the Institutional Review Board (IRB) (approval no.: AMC/PGMI/156/1/2023). The study enrolled 356 patients over a one-year period from March 2023 to February 2024. Prior to participation, informed consent was obtained from all subjects after a thorough explanation of the study's purpose and assurance of adherence to ethical considerations, including patient confidentiality and the right to withdraw without consequences.

This study included adults aged 18-65 years with diagnosed CKD stages I-V, having disease duration of at least three months and not dialysis dependent. We excluded patients on dialysis, those with kidney transplants, acute kidney injury, documented ischemic heart disease, congenital or significant valvular heart diseases, primary cardiomyopathies, atrial fibrillation, pacemakers, history of heart transplantation or mitral valve surgery, moderate to severe mitral valve disease or annular calcification, pregnancy, intellectual disability, psychosis and active malignancy. We also excluded patients with incomplete echocardiographic studies or those unwilling to provide informed consent. The stable CKD stage was confirmed by two consecutive serum creatinine measurements within three months. Baseline demographic and clinical details were collected.

Echocardiography data from CKD patients were reviewed for reports performed within the last one month; those without recent reports were referred to a consultant cardiologist for echocardiographic evaluation. The comprehensive echocardiographic assessment included left ventricular hypertrophy (LVH), ejection fraction (EF), diastolic dysfunction, left ventricular systolic dysfunction categorized as normal (EF ≥ 55%), borderline (45-55%), mild (35-44%), moderate (25-34%) and severe (< 25%), global longitudinal strain (GLS), valvular function (mitral and tricuspid regurgitation severity), and pulmonary hypertension. Quantitative measurements included left ventricular ejection fraction (LVEF), left ventricular mass index (LVMI), left ventricular end-diastolic diameter (LVEDD), left ventricular end-systolic diameters (LVESD), left atrial volume index (LAVI), relative wall thickness (RWT), A/E ratio, pulmonary artery systolic pressure (PASP), and tricuspid regurgitation (TR) velocity. This thorough protocol ensured a detailed evaluation of the cardiac structure and function across various parameters relevant to CKD patients. Data was recorded using preformed data collection proforma.

Statistical analysis was performed using Statistical Package for the Social Sciences (IBM SPSS Statistics for Windows, IBM Corp., Version 26.0, Armonk, NY). Quantitative variables were expressed as mean and standard deviation, while qualitative variables were presented as frequencies and percentages. To assess differences across CKD stages (I, II, III, IV, and V), one-way analysis of variance (ANOVA) was employed for quantitative variables and Chi-square or Fisher's exact tests were used for qualitative variables as appropriate. A p-value <0.05 was considered statistically significant for all comparisons.

## Results

This study included 356 patients with CKD across stages I-V. The majority of patients were in stages III (44.7%) and IV (36.5%). There were no significant differences in gender distribution or prevalence of diabetes across CKD stages. However, age (p<0.000), hypertension prevalence (p=0.004), and smoking status showed significant variations. Laboratory parameters including haemoglobin, calcium, and phosphate levels differed significantly across stages (all p<0.000), with haemoglobin and calcium decreasing and phosphate increasing as CKD progressed (Table 1).

**Table 1:** Baseline characteristics of patients across different stages of chronic kidney disease Hemoglobin level (g/dL): Reference range: 13.5-17.5 g/dL (males), 12.0-15.5 g/dL (females) Calcium level (mg/dL): Reference range: 8.5-10.2 mg/dL Phosphate level (mg/dL): Reference range: 2.5-4.5 mg/dL One-way analysis of variance (ANOVA) was applied to quantitative variables. The chi-square test was applied to qualitative variables. GFR: glomerular filtration rate

Baseline Characteristics	Stages I-II 32 (9%)	Stage III 159 (44.7%)	Stage IV 130 (36.5%)	Stage V 35 (9.8%)	p-value
Gender (n, %)					
Male	13 (40.6%)	67 (42.1%)	47 (36.2%)	20 (57.1%)	0.165
Female	19 (59.4%)	92 (57.9%)	83 (63.8%)	15 (42.9%)	
Age (years)	37.41 ± 11.10	38.77 ± 14.32	41.95 ± 13.27	52.54 ± 7.66	0.000
Hypertension (n, %)	11 (34.4%)	83 (52.2%)	74 (56.9%)	27 (77.1%)	0.004
Diabetes (n, %)	11 (34.4%)	64 (40.3%)	65 (50.0%)	12 (34.3%)	0.162
Hyperlipidemia (n, %)	10 (31.2%)	80 (50.3%)	72 (55.4%)	25 (71.4%)	0.009
Smoking Status (n, %)					0.786
Active Smoker	7 (21.9%)	38 (23.9%)	25 (19.2%)	9 (25.7%)	
Former Smoker	7 (21.9%)	35 (22.0%)	38 (29.2%)	10 (28.6%)	
Non-smoker	18 (56.2%)	86 (54.1%)	67 (51.5%)	15 (45.7%)	
Haemoglobin Level (g/dL)	12.27 ± 1.25	10.78 ± 1.22	9.59 ± 1.33	9.44 ± 0.94	0.000
Calcium Level (mg/dL)	8.74 ± 0.21	8.19 ± 0.43	8.09 ± 0.38	7.69 ± 0.51	0.000
Phosphate Level (mg/dL)	4.25 ± 0.41	4.43 ± 0.55	5.52 ± 0.91	5.33 ± 0.98	0.000
Estimated GFR (mL/min/1.73 m²)	75.67 ± 8.81	44.06 ± 7.38	22.81 ± 3.85	11.31±2.14	0.000

The echocardiographic findings revealed significant differences across CKD stages. LVH prevalence increased from 12.5% in stages I-II to 60.0% in stage V (p=0.001). Diastolic dysfunction worsened with CKD progression, with grades 2-3 dysfunction rising from 6.2% in stages I-II to 51.4% in stage V (p=0.000). Systolic dysfunction also increased, with reduced EF (<45%) more common in advanced stages (p=0.000). GLS worsened from -18.47±1.10% in stages I-II to -15.34±2.21% in stage V (p=0.000). LVEF decreased from 63.03±5.53% to 41.69±8.65% (p=0.000), while the LVMI increased from 82.34±10.08 g/m² to 109.37±15.73 g/m² (p=0.000). The LAVI rose from 26.69±3.32 mL/m² to 38.54±5.01 mL/m² (p=0.000). Mitral and TR prevalence and severity increased in advanced stages (both p=0.000). Pulmonary hypertension prevalence increased from 18.8% in stages I-II to 51.4% in stage V (p=0.049). PASP rose from 29.63±4.51 mmHg to 38.94±6.17 mmHg (p=0.000). These findings demonstrate progressive cardiac structural and functional changes correlating with CKD severity (Table 2).

**Table 2:** Echocardiographic characteristics of patients across different stages of chronic kidney disease One-way analysis of variance (ANOVA) was applied to quantitative variables. The chi-square test was applied to qualitative variables. LVH: left ventricular hypertrophy; EF: ejection fraction; LVEDD: left ventricular end-diastolic diameter; LVESD: left ventricular end-systolic diameter

Echocardiographic Characteristics	Stages I-II 32 (9%)	Stage III 159 (44.7%)	Stage IV 130 (36.5%)	Stage V 35 (9.8%)	p-value
LVH (n, %)	4 (12.5%)	74 (46.5%)	57 (43.8%)	21 (60.0%)	0.001
Grades of Diastolic Dysfunction (n, %)					0.000
Normal	28 (87.5%)	70 (44.0%)	33 (25.4%)	9 (25.7%)	
Grade 1	2 (6.2%)	30 (18.9%)	43 (33.1%)	8 (22.9%)	
Grade 2	2 (6.2%)	46 (28.9%)	40 (30.8%)	13 (37.1%)	
Grade 3	0 (0.0%)	13 (8.2%)	14 (10.8%)	5 (14.3%)	
Grades of Systolic Dysfunction (n, %)					0.000
Normal (EF > 55%)	28 (87.5%)	61 (38.4%)	52 (40.0%)	4 (11.4%)	
Borderline (EF 45-55%)	4 (12.5%)	51 (32.1%)	50 (38.5%)	7 (20.0%)	
Mild (EF 35-44%)	0 (0.0%)	42 (26.4%)	20 (15.4%)	16 (45.7%)	
Moderate (EF 25-34%)	0 (0.0%)	5 (3.1%)	8 (6.2%)	8 (22.9%)	
Global Longitudinal Strain (n, %)	2 (6.2%)	56 (35.2%)	52 (40.0%)	22 (62.9%)	0.000
Mitral Regurgitation Murmur (n, %)					0.000
Absent	30 (93.8%)	110 (69.2%)	92 (70.8%)	16 (45.7%)	
Mild	2 (6.2%)	27 (17.0%)	26 (20.0%)	7 (20.0%)	
Moderate	0 (0.0%)	22 (13.8%)	11 (8.5%)	6 (17.1%)	
Severe	0 (0.0%)	0 (0.0%)	1 (0.8%)	6 (17.1%)	
Tricuspid Regurgitation Murmur (n, %)					0.000
Absent	30 (93.8%)	113 (71.1%)	99 (76.2%)	22 (62.9%)	
Mild	2 (6.2%)	35 (22.0%)	4 (3.1%)	1 (2.9%)	
Moderate	0 (0.0%)	11 (6.9%)	26 (20.0%)	10 (28.6%)	
Severe	0 (0.0%)	0 (0.0%)	1 (0.8%)	2 (5.7%)	
Pulmonary Hypertension (n, %)	6 (18.8%)	61 (38.4%)	52 (40.0%)	18 (51.4%)	0.049
Left Ventricular Ejection Fraction (%)	63.03 ± 5.53	52.52 ± 7.03	48.43 ± 7.39	41.69 ± 8.65	0.000
Left Ventricular Mass Index (g/m²)	82.34 ± 10.08	98.57 ± 15.39	99.45 ± 13.66	109.37 ± 15.73	0.000
LVEDD (mm)	48.37 ± 4.94	53.17 ± 4.41	52.45 ± 6.47	56.60 ± 7.15	0.000
LVESD (mm)	35.09 ± 6.02	42.34 ± 4.61	42.69 ± 8.47	43.37 ± 6.06	0.000
Left Atrial Volume Index (mL/m²)	26.69 ± 3.32	31.35 ± 4.04	37.09 ± 4.74	38.54 ± 5.01	0.000
Relative Wall Thickness	0.40 ± 0.02	0.43 ± 0.03	0.44 ± 0.04	0.45 ± 0.04	0.000
Global Longitudinal Strain (%)	-18.47 ± 1.10	-17.09 ± 1.14	-16.85 ± 1.75	-15.34 ± 2.21	0.000
A/E Ratio	1.10 ± 0.15	1.14 ± 0.10	1.75 ± 0.38	1.81 ± 0.25	0.000
Pulmonary Artery Systolic Pressure (mmHg)	29.63 ± 4.51	33.26 ± 4.23	39.71 ± 4.86	38.94 ± 6.17	0.000
Tricuspid Regurgitation Velocity (m/s)	2.65 ± 0.44	2.92 ± 0.27	2.87 ± 0.27	2.74 ± 0.25	0.000

## Discussion

CVD significantly affects the prognosis of CKD. Concurrently, CKD is recognized for its impact on cardiac structural and functional abnormalities. Therefore, early detection of cardiac abnormalities is crucial for timely intervention in CKD to improve patient outcomes. This study included 356 non-dialysis-dependent CKD patients, with most being in CKD stage III (44.7%), followed by stage IV (36.5%). There was a lower proportion of patients in CKD stages I and II (9%), which may be attributed to late presentation to the nephrologist. Another study reported a high proportion of stage IV CKD patients [11].

In our study, there were more female patients (58.7%) compared to males (41.3%). This is in contrast to previous studies, which reported a higher prevalence of male patients compared to female patients [7,12,13]. Most of the patients in the current study were between the ages of 45 and 65 years. Routray et al. (2024) reported that the majority of their patients were in the age group of 60-75 years, a similar age group to that reported in previous studies [10-12].

In this study, hypertension was the most prevalent comorbidity, affecting 54.8% of the patients, followed by hyperlipidemia in 52.5% and diabetes in 42%. Similarly, a recent study reported hypertension as the most prevalent co-morbidity [11]. In contrast, Ansari et al. (2024) reported a higher proportion of diabetic patients [12].

The prevalence of LVH in our study was 43.8%, with the proportion increasing as the stage of CKD advanced. Stages I and II had an LVH frequency of 12.5%, which increased to 60% in stage IV non-dialysis-dependent CKD. This trend demonstrated a positive correlation between CKD stages and cardiac abnormalities, supporting findings from previous studies in non-dialysis-dependent CKD patients. Ansari et al. (2024) reported an overall LVH prevalence of 60% in non-dialysis-dependent patients [12]. Routray et al. (2024) found that LVH increased from 64% in stage II CKD to 93% in stage IV CKD, which is comparatively higher than the current study [11]. Another study reported an overall LVH prevalence of 80% in the CKD population, with a prevalence of 15% in stages I and II, rising to 47% in stage V [7].

In this study, 60% of the patients had mild to moderate systolic and diastolic dysfunction, which advanced with the stages of CKD. A recent study by Olsen et al. (2024) evaluated the effect of estimated glomerular filtration rate (eGFR) on the global work index of the heart and reported a direct association between lower eGFR and reduced global work index (GWI) [14].

In the study, the LVMI increased from 82.34±10.08 g/m² to 109±15.73 g/m² from stage I to stage V CKD. Similarly, LVEDD worsened from 48±4.1 mm to 56.6±7.15 mm, and LVESD deteriorated from 35±9 mm to 43.3±6.06 mm. GLS deteriorated from -18% to -15% with the progression of CKD stages (p-value <0.001). Similarly, all other quantitative echocardiography parameters reported statistically significant deterioration as advancing CKD stages. A study by Routray et al. (2024) reported a greater decline in these parameters compared to the current study. They found that GLS deteriorated from -16% to -12% and TR velocity increased from 2.19±0.04 m/s to 2.83±0.07 m/s (p-value <0.001). The greater decline in their study may be due to differences in the enrolled participants, as they also included patients aged over 65 years and had more stage V CKD patients compared to our study [11].

Liang et al. also reported significant deterioration in echocardiographic parameters similar to our study. LVMI increased from 82 to 105 g/m², LAVI increased from 27 to 38 mL/m², and TR velocity increased from 2.2±0.47 m/s to 2.62±0.62 m/s [7]. The progressive deterioration of LAVI and LVMI is often found as a result of chronic pressure and volume overload [15,16]. Nardi et al. (2019) compared the echocardiographic findings of non-dialysis-dependent CKD patients and hypertensive patients. They found that systolic dysfunction was 70% in CKD patients compared to 40% in hypertensive patients without CKD. There was also a significant correlation between diastolic dysfunction and decreasing eGFR (p-value < 0.001) [6].

This study's strengths include a detailed echocardiographic evaluation including various structural and functional cardiac parameters in a substantial sample size of non-dialysis-dependent CKD patients. However, its cross-sectional nature limits the ability to establish causal relationships, and the exclusion of dialysis-dependent patients may affect the general applicability of these findings. Future multicenter studies should include samples from different populations and compare the echocardiographic findings between non-dialysis CKD patients and patients receiving different modalities of dialysis, such as peritoneal and hemodialysis, for a better understanding of cardiac remodelling and the effect of CKD. Additionally, incorporating advanced imaging techniques could offer deeper insights into cardiac alterations associated with CKD progression.

## Conclusions

The study reveals worsening of echocardiographic abnormalities with advancing stages of CKD. Notably, LVH frequency substantially increased with CKD progression. Both systolic and diastolic dysfunctions, along with other echocardiographic parameters, showed marked deterioration with CKD progression. These results emphasize the need for early and routine cardiac assessments in patients with early CKD stages to identify and manage cardiovascular risks promptly. Early detection and timely management of cardiac issues in CKD patients can significantly improve their overall prognosis.
